# Translation and cross-cultural adaptation of the ICHOM standard set for stroke: the Dutch version

**DOI:** 10.1186/s41687-023-00630-7

**Published:** 2023-09-11

**Authors:** Daniëlla M Oosterveer, Winke van Meijeren-Pont, Frederike van Markus-Doornbosch, Etienne Stegeman, Caroline B Terwee, Gerard M Ribbers, Thea PM Vliet Vlieland

**Affiliations:** 1 Basalt, Wassenaarseweg 501, 2333 AL, Leiden/The Hague, The Netherlands; 2 Department of Orthopaedics, Rehabilitation and Physical Therapy, Leiden University Medical Center, Leiden, The Netherlands; 3 Alrijne, Department of Rehabilitation Medicine, Leiden, The Netherlands; 4 Department of Epidemiology and Data Science, Amsterdam UMC, Vrije Universiteit Amsterdam, Amsterdam, The Netherlands; 5 Amsterdam Public Health Research Institute - Methodology, Amsterdam, The Netherlands; 6 Department of Rehabilitation, Erasmus University Medical Center, Rotterdam, The Netherlands; 7 Rijndam Rehabilitation Center, Rotterdam, The Netherlands

**Keywords:** Stroke, ICHOM, Translation, Dutch, Standard Set for Stroke

## Abstract

**Introduction:**

The International Consortium for Health Outcomes Measurement (ICHOM) developed a standard set of patient-centered outcome measures for use in stroke patients. In addition to the Patient-Reported Outcomes Measurement Information System (PROMIS) Global Health, it is comprised of 25 questions that are not part of a specific questionnaire. This study aimed to translate these 25 single questions into Dutch.

**Methods:**

Two native Dutch-speaking translators independently translated the original ICHOM questions into Dutch. A consensus translation was made by these translators and a third person. This translation was subsequently translated back to English independently by two native English-speaking translators. Afterwards a pre-final version was made by consensus of a committee. After field-testing among 30 stroke patients, a final version was made.

**Results:**

The forward and backward translations led to eight cross-cultural adaptations. Based on the interviews with stroke patients, 12 questions were changed to enhance comprehensibility leading to a final Dutch translation of the 25 single questions.

**Conclusions:**

A Dutch translation of the 25 single questions of the ICHOM Standard Set for Stroke was developed. Now a complete ICHOM Standard Set for Stroke can be used in Dutch populations allowing comparison and improvement of stroke care.

**Supplementary Information:**

The online version contains supplementary material available at 10.1186/s41687-023-00630-7.

## Introduction

Stroke is the second leading cause of death and of disability worldwide [1]. From 1990 to 2017, the absolute number of people who suffered a new stroke has almost doubled [1]. Stroke can lead to a multitude of impairments such as motor impairments [2], aphasia [3], cognitive problems [4], fatigue [5] and depression [6], resulting in a diminished Quality of Life (QoL) [7].

This wide range of sequelae is assessed with a large number of different disease-specific and general outcome measures in stroke health care and research. This lack of consensus of outcome measures limits benchmarking of the quality of stroke health care and interpretation of outcomes of stroke research [8].

Therefore, the International Consortium for Health Outcomes Measurement (ICHOM) developed a Standard Set for Stroke using a structured consensus-driven modified Delphi method with an international expert panel [9]. The ICHOM Standard Set for Stroke consists of a combination of case mix variables, treatment variables and outcomes. These data are collected using administrative data, clinical data and Patient-Reported Outcome Measures (PROMs). The PROMs consist of 25 single questions and the Patient-Reported Outcomes Measurement Information System (PROMIS®) Global Health [9]. Currently, the first studies reporting on the use of this standard set for stroke have been published [10–12]. In addition, a benchmarking platform was set up by ICHOM allowing facilities to add and compare the data from this Standard Set for Stroke (https://www.ichom.org/global-benchmarking-platform/).

To allow a valid interpretation and international comparison of the ICHOM Standard Set for Stroke, adequate translations of the PROMs of this set are needed. The PROMIS Global Health is already translated in several languages including Dutch. This Dutch translation was shown to have sufficient psychometric properties in the Dutch population [13]. The aim of our study was to also translate the 25 single questions of this Standard Set for Stroke in Dutch so that a complete Standard Set for Stroke can be used in Dutch populations.

## Materials and methods

### Design

This study is a translation and cross-cultural adaptation study including pilot testing among patients with stroke. The translation and pilot testing process were based on previously developed guidelines for cross-cultural translation of PROMs to ensure a comprehensible translation equivalent to the original questions [14–16]. For the pilot testing, patients who participated in the Stroke Cohort Outcomes of Rehabilitation (SCORE) 2.1 study (Netherlands Trial Register no. NL9509) were recruited. The study protocol was reviewed by the Medical Ethical Committee of the Leiden University Medical Center and was found not to fall within the scope of the Dutch Medical Research with Human Subjects Law (N21.070). All patients signed informed consent and an additional informed consent for the interview. The study was conducted in compliance with the Declaration of Helsinki [17]. ICHOM was notified about this translation study and had no objection to the study.

### The 25 questions of the ICHOM standard set for stroke

The 25 questions of the ICHOM standard set for stroke consist of 8 questions about ‘demographic factors’, 10 about ‘vascular and systemic’ history, 2 about ‘survival and disease control’ and 5 about ‘patient-reported health status’. ICHOM has assigned a variable ID to each question to facilitate benchmarking. The question concerning ethnicity should be formulated by each country and is not predefined as a single question. All other questions are multiple-choice questions.

### Translation of the questions of the ICHOM standard set for stroke

First, the English questions were independently translated into Dutch by two native Dutch-speaking translators (DO, WvMP), who are fluent in English and experienced in the field of stroke research.

Second, a meeting was organized with these translators and a third native Dutch-speaking medical professional (GR). This medical professional was also involved in the development of the original questions. In this meeting the two forward translations were reconciled. Discrepancies were resolved or the better of the forward translations was chosen. In case of doubt, the ICHOM was contacted.

Thirdly, after this consensus Dutch translation was developed, the translation was translated back into English by two native English-speaking translators (FvM, EP), who were also fluent in the Dutch language. One of the native English-speaking translators was experienced in the field of PROMs and the other native English-speaking translator was not involved in research or PROMs. Both were blinded to the original English questions.

Fourth, a committee (comprised of the translation team, and two additional medical professionals) compared the two back-translations with the original 25 questions and provided a clarification for the differences. An alternate Dutch translation was made when the previous translation was not acceptable based on the back-translations. This resulted in the pre-final version.

### Pilot testing of the translated questions of the ICHOM standard set for stroke

The pre-final version that resulted from above described translation process was tested for comprehensibility in a sample of 30 stroke patients [18]. All patients were aged 18 or older and admitted to a rehabilitation center for inpatient stroke rehabilitation. Both patients with a first-ever stroke as with a recurrent stroke were included. Patients were excluded when they were unable to complete questionnaires in Dutch. Patients with aphasia or cognitive problems were only excluded if they were not able to answer the questions and participate in the interview. These patients completed the pre-final version of the 25 Dutch questions. Afterwards, they were interviewed by a research nurse to ensure that the substance of information of the translated question remained the same as the original English question and that the questions were comprehensible. After the interviews, the patient’ comments were analyzed and discussed, and when needed adjustments were made by the committee, resulting in the final translation.

## Results

### Translation

The original questions and their variable IDs are shown in Table 1. After the two forward translations were completed, a consensus meeting with a Dutch-speaking medical professional was organized, resulting in the identification of eight cross-cultural issues. In the questions concerning living location (i.e. variable IDs LIVINGLOCPRE and LIVINGLOCPOST) specific health care facilities are mentioned that are not available in the Netherlands. Thus, we incorporated equivalent facilities in the Dutch health care system in these questions. As for LIVINGLOCPRE there was additional e-mail contact with the ICHOM, because ‘living’ in Dutch suggests a permanent stay and one of the original response options (i.e. option 4) is not a permanent living location in the Netherlands. Based on the advice from ICHOM, consensus was reached on adding another question (LIVINGLOCREHAB) when option 4 was chosen to identify the patients permanent living location pre-stroke. In addition, there was discussion about what is included within community care in option 1 and 2 of LIVINGLOCPRE and LIVINGLOCPOST. There is no direct Dutch translation for this term. ICHOM clarified this as being professional care, and the translation was adjusted accordingly.

**Table 1:** Original English version and final Dutch version of the ICHOM questions of the Standard Set for Stroke

Variable (Variable ID)	Original Question ICHOM	Final Dutch Translation
**Demographic factors**		
Ethnicity (ETHNIC)^a^	Varies by country and should be determined by country (not for cross country comparison)	In welk land bent u geboren? In welk land is uw biologische vader geboren? In welk land is uw biologische moeder geboren?
Living location pre index event (LIVINGLOCPRE)^a^	Where were you living prior to your stroke or transient ischaemic attack (TIA)? 1 = At home, with no community support 2 = At home with community support 3 = In an assisting living home in the community (senior’s home) 4 = In a rehabilitation hospital or skilled care facilities (SNIF, IRF, LTACH) 5 = In long term care (nursing home, chronic care hospital) 888 = Other 999 = Unknown	Waar verbleef u voordat u een beroerte of TIA kreeg? 1 = Thuis zonder hulp van thuiszorg en/of buurtzorg 2 = Thuis met hulp van thuiszorg en/of buurtzorg 3 = In een aanleunwoning, woonzorgcentrum of focuswoning 4 = In een revalidatiecentrum, in een zorghotel of een revalidatieafdeling in een verpleeghuis 5 = Op een verblijfsafdeling in een verpleeghuis 888 = Anders 999 = Onbekend *LIVINGLOCREHAB* *Indien u antwoord 4 heeft gegeven: waar woonde u voordat u in een revalidatiecentrum, in een zorghotel of een revalidatieafdeling in een verpleeghuis was opgenomen?* *1 = Thuis zonder hulp van thuiszorg en/of buurtzorg* *2 = Thuis met hulp van thuiszorg en/of buurtzorg* *3 = In een aanleunwoning, woonzorgcentrum of focuswoning* *5 = Op een verblijfsafdeling in een verpleeghuis* *888 = Anders* *999 = Onbekend*
Living location post index event (LIVINGLOCPOST)^c^	Where are you living now? 1 = At home, with no community support 2 = At home with community support 3 = In an assisting living home in the community (senior’s home) 4 = In a rehabilitation hospital or skilled care facilities (SNIF, IRF, LTACH) 5 = In long term care (nursing home, chronic care hospital 6 = In an acute care hospital 888 = Other 999 = Unknown	Waar verblijft u op dit moment? 1 = Thuis zonder hulp van thuiszorg en/of buurtzorg 2 = Thuis met hulp van thuiszorg en/of buurtzorg 3 = In een aanleunwoning, woonzorgcentrum of focuswoning 4 = In een revalidatiecentrum, in een zorghotel of een revalidatieafdeling in een verpleeghuis 5 = Op een verblijfsafdeling in een verpleeghuis 6 = In een ziekenhuis 888 = Anders 999 = Onbekend
Living alone pre-index event (LIVEALONEPRE)^a^ If “1 = At home, with no community support”	Did you live alone prior to your stroke or transient ischaemic attack (TIA)? 1 = Yes, I lived alone 2 = No, I shared my household with spouse/partner or other person (e.g. sibling, children, parents) 999 = Unknown	Woonde u alleen voordat u een beroerte of TIA kreeg? 1 = Ja, ik woonde alleen 2 = Nee, ik woonde samen met mijn partner en/of andere personen (zoals kinderen, broers, zussen, ouders) 999 = Onbekend
Living alone post-index event (LIVEALONEPOST)^c^	Do you live alone now? 1 = Yes, I live alone 2 = No, I share my household with spouse/partner or other person (e.g. sibling, children, parents) 999 = Unknown	Woont u alleen op dit moment? 1 = Ja, ik woon alleen 2 = Nee, ik woon samen met mijn partner en/of andere personen (zoals kinderen, broers, zussen, ouders) 999 = Ik verblijf op dit moment niet thuis of onbekend
Prestroke functional status – Ambulation (PRESTROKEAMB)^a^	Were you able to walk prior to your stroke or transient ischaemic attack (TIA)? 1 = Able to walk without help from another person with or without a device 2 = Able to walk with help from another person 3 = Unable to walk	Kon u lopen voordat u een beroerte of TIA kreeg? 1 = Ja, ik kon zelfstandig lopen (met of zonder hulpmiddel) 2 = Ja, ik kon lopen met hulp van een persoon (met of zonder hulpmiddel) 3 = Nee, ik kon niet lopen
Prestroke functional status – Toileting (PRESTROKETOILET)^a^	Did you need help from anybody to go to the toilet prior to your stroke or transient ischaemic attack (TIA)? 1 = I could manage going to the toilet without assistance 2 = I needed help to go to the toilet	Kon u zelfstandig naar het toilet gaan voordat u een beroerte of TIA kreeg? 1 = Ja, ik kon zelfstandig naar het toilet gaan 2 = Nee, ik had hulp van iemand nodig om naar het toilet te gaan
Prestroke functional status – Dressing (PRESTROKEDRESS)^a^	Did you need help with dressing/undressing prior to your stroke or transient ischaemic attack (TIA)? 1 = I could manage dressing/undressing without help 2 = I needed help dressing/undressing	Kon u zichzelf aan- en uitkleden voordat u een beroerte of TIA kreeg? 1 = Ja, ik kon mijzelf zelfstandig aan- en uitkleden 2 = Nee, ik had hulp nodig bij het aan- en uitkleden
**Vascular and Systemic** All these items are phrased as a patient reported measure. However, if the patient is unable to answer, this information can be abstracted from the medical records.		
Prior Stroke (PRIORSTROKE)^a^	Prior to this hospitalization, have you ever been told by a doctor that you have had a stroke? 0 = No 1 = Yes 999 = Unknown	Heeft een dokter voor deze beroerte ooit eerder bij u een beroerte vastgesteld? 0 = Nee 1 = Ja 999 = Ik weet het niet
Prior TIA (PRIORTIA)^a^	Have you ever been told by a doctor that you have had a transient ischemic attack (this is sometimes called a TIA or mini-stroke)? 0 = No 1 = Yes 999 = Unknown	Heeft een dokter ooit bij u een TIA vastgesteld? 0 = Nee 1 = Ja 999 = Ik weet het niet
Prior MI (PRIORMI)^a^	Have you ever been told by your doctor that you’ve had a heart attack (this is sometimes called a myocardial infarction, or MI)? 0 = No 1 = Yes 999 = Unknown	Heeft een dokter ooit bij u een hartaanval vastgesteld? 0 = Nee 1 = Ja 999 = Ik weet het niet
Coronary artery disease (CAD)^a^	Have you ever been told by your doctor that you have coronary artery disease? 0 = No 1 = Yes 999 = Unknown	Heeft een dokter ooit bij u coronaire hartziekten vastgesteld (ook wel angina pectoris genoemd)? *Coronaire hartziekte is een aandoening, waarbij de bloedtoevoer naar de hartspier belemmerd wordt.* 0 = Nee 1 = Ja 999 = Ik weet het niet
Atrial fibrillation (AFIB)^a^	Have you ever been told by your doctor that you have atrial fibrillation? 0 = No 1 = Yes 999 = Unknown	Heeft een dokter ooit bij u boezemfibrilleren vastgesteld (ook wel atriumfibrilleren genoemd)? *Boezemfibrilleren of atriumfibrilleren is een hartritmestoornis, waarbij de hartslag onregelmatig en meestal te hoog is.* 0 = Nee 1 = Ja 999 = Ik weet het niet
Diabetes mellitus (DIAB)^a^	Have you ever been told by your doctor that you have diabetes? 0 = No 1 = Yes 999 = Unknown	Heeft een dokter ooit bij u suikerziekte vastgesteld (ook wel diabetes genoemd)? 0 = Nee 1 = Ja 999 = Ik weet het niet
Hypertension (HYPERTENS)^a^	Have you ever been told by a doctor that you have high blood pressure (this is sometimes called hypertension)? 0 = No 1 = Yes 999 = Unknown	Heeft een dokter ooit bij u een hoge bloeddruk vastgesteld (ook wel hypertensie genoemd)? 0 = Nee 1 = Ja 999 = Ik weet het niet
Hyperlipidemia (HYPERLIP)^a^	Have you ever been told by your doctor that you have high cholesterol (this is sometimes called hyperlipidemia or dyslipidemia)? 0 = No 1 = Yes 999 = Unknown	Heeft een dokter of een verpleegkundige/doktersassistent ooit bij u een hoog cholesterol vastgesteld? 0 = Nee 1 = Ja 999 = Ik weet het niet
Smoking status (SMOKE)^a^	Do you currently smoke, or have you smoked cigarettes or tobacco over the past year? 0 = No 1 = Yes 999 = Unknown	Rookt u op dit moment of heeft u het afgelopen jaar gerookt? 0 = Nee 1 = Ja 999 = Onbekend
Alcohol use (ALCOHOL)^a^	Do you drink more than one alcoholic drink a day? 0 = No 1 = Yes 999 = Unknown	Drinkt u meer dan 1 glas alcohol per dag? 0 = Nee 1 = Ja 999 = Onbekend
**Survival and Disease controle**		
Report of new stroke within 90 days after admission for stroke (STROKERECUR)^c^	After your hospitalization for stroke, have you been told by a doctor that you have had a new stroke? 0 = No 1 = Yes 999 = Unknown	U heeft een beroerte gehad. Heeft een dokter bij u daarna opnieuw een beroerte vastgesteld? 0 = Nee 1 = Ja 999 = Ik weet het niet
Smoking cessation (SMOKECESS)^c^ If “1 = Yes” to SMOKE	Since your hospitalization for stroke, have you smoked tobacco or cigarettes? 0 = No 1 = Yes 999 = Unknown	Heeft u gerookt na uw beroerte? 0 = Nee 1 = Ja 999 = Onbekend
**Patient-Reported Health Status**		
Poststroke functional status – Ambulation (POSTSTROKEAMB)^b,c^	Are you able to walk? 1 = Able to walk without help from another person with or without a device 2 = Able to walk with help from another person 3 = Unable to walk	Kunt u lopen? 1 = Ja, ik kan zelfstandig lopen (met of zonder hulpmiddel) 2 = Ja, ik kan lopen met hulp van een persoon (met of zonder hulpmiddel) 3 = Nee, ik kan niet lopen
Poststroke functional status – Toileting (POSTSTROKETOILET)^b,c^	Do you need help from anybody to go to the toilet? 1 = I can manage going to the toilet without assistance 2 = I need help to go to the toilet	Kan u zelfstandig naar het toilet te gaan? 1 = Ja, ik kan zelfstandig naar het toilet gaan 2 = Nee, ik heb hulp van iemand nodig om naar het toilet te gaan
Poststroke functional status – Dressing (POSTSTROKEDRESS)^b,c^	Do you need help with dressing/undressing? 1 = I can manage dressing/undressing without help 2 = I need help dressing/undressing	Kan u zichzelf aan- en uitkleden? 1 = Ja, ik kan mijzelf zelfstandig aan- en uitkleden 2 = Nee, ik heb hulp nodig bij het aan- en uitkleden
Feeding (FEEDING)^b,c^	Do you need a tube for feeding? 0 = No 1 = Yes	Heeft u een sonde nodig om te eten, b.v. een neussonde of een maagsonde? 0 = Nee 1 = Ja
Ability to communicate (COMMUNIC)^b,c^	Do you have problems with communication or understanding? 0 = No 1 = Yes	Heeft u problemen met praten of begrijpen van taal? 0 = Nee 1 = Ja

^a^Timing question: admission for index event

^b^Timing question: Discharge + 7 days

^c^Timing question: 90 days post admission for index event

For the translation of the questions concerning vascular and systemic medical history, the phrase or phrases for each medical condition were used that were thought to be most commonly used in the general Dutch population and therefore most comprehensible for Dutch stroke patients. For example, mini-stroke is not commonly used and was left out of the translation of question PRIORTIA, while we added the word ‘suikerziekte’ (‘sugar disease’) to diabetes in question DIAB. In addition, we chose to consistently use ‘a’ doctor, while in the original questions ‘your’ doctor and ‘a’ doctor are used inconsistently. In addition, a more free translation of the response option ‘999 = Unknown’, for the questions about medical history was chosen. This was done to give patients the option that they do not know the answer as an alternative for leaving the question unanswered.

After backward translation, a meeting with the committee took place. This led to the identification of an error in the two questions concerning ambulation (PRESTROKEAMB and POSTSTROKEAMB), which was corrected accordingly. A pre-final version was made. In Supplementary Tables 1, the translations resulting from each above mentioned step are described.

### Pilot testing

The pre-final version of the ICHOM questions of the Standard Set for Stroke was tested in 30 stroke patients for comprehensibility. The mean age of these patients was 60 years (range 38–83 years), 30% of them were females, 90% had an ischemic stroke. The interviews with these patients after completing the questions lead to changes in the wording of 12 questions.

The question LIVEALONEPOST should be completed by all patients, but most of our patients that were admitted to the rehabilitation center found it difficult to answer as they did not reside at home when filling in the questionnaire. To overcome this difficulty, option 999 was changed from ‘Unknown’ into ‘I am not living at home at the moment or unknown’. The questions concerning prestroke and poststroke functional status would be easier to answer when response options of these six questions would start with ‘yes’ and ‘no’ according to 6–8 patients depending on the question. This was done accordingly.

The Dutch equivalents for the terms coronary artery disease and atrial fibrillation were unknown to 40% and 30% of patients, respectively. We therefore added an explanation of these terms to the questions concerning these diseases (i.e. CAD and AFIB). Some patients reported that they had high cholesterol but that this was mentioned by a nurse or an assistant of their doctor and not by their doctor. Because this is common practice in the Netherlands, the question HYPERLIP was changed accordingly. The questions PRIORSTROKE and STROKERECUR were not always clear for patients and were clarified. During the discussion of the interviews of the patients, a better suited translation of the word ‘told’ in the questions about medical conditions was found and applied accordingly. The original questions and the final Dutch version of the 25 single questions in the ICHOM Standard Set for Stroke is shown in Table 1.

## Discussion

This cross-cultural translation study of 25 questions of the ICHOM Standard Set for Stroke into Dutch resulted in a comprehensible translation equivalent to the original questions. Equivalence is necessary to allow international comparison between national stroke populations and to allow pooling of data collected from stroke patients in different countries [16]. Thereby, this translation contributes to the aims of the ICHOM to effectively improve stroke health care and usability of research.

The value of an international standard set has been shown previously with the Utstein template for patients with cardiac arrest [19]. This template has been widely implemented and used for international comparison of cardiopulmonary resuscitation outcomes [20, 21]. Similar to the ICHOM Standard Set of Stroke, the Utstein template includes amongst others case mix variables and outcomes. However, unlike the ICHOM Standard Set for Stroke, it does not include PROMs.

As the authors of the ICHOM Standard Set for Stroke also state themselves, the utility of the Standard Set in clinical practice and research is still undetermined, because it is based on expert consensus rather than on high levels of evidence. The original single questions were not tested for comprehensibility in stroke patients nor is there a specific lay-out. In addition, to our knowledge no other translation of the complete Standard Set for Stroke exists, hampering harmonization and international comparison. A recent study demonstrated that both patients and medical staff found the Standard Set for Stroke relevant, but patients reported limited understanding of why the assessment was introduced and the medical staff found limited feasibility and sustainability of the set [12]. Their acceptance of the implementation were low [12]. Moreover, de Graaf et al. [22] found that factors not related to the stroke (such as coping style) influence the scores on PROMIS Global Health. This emphasized the need for further research.

Nevertheless, there is increasing evidence that the measurement properties of the PROMIS Global Health are favorable in hospital-based stroke populations and in general populations [23, 24]. In addition, in the Dutch population, the measurement properties of the Dutch-Flemish PROMIS Global Health were also favorable [13] and reference values are available [25].

### Limitations

Our study focused on comprehensibility, however further research is necessary to assess the other psychometric properties of the translated ICHOM Standard Set for Stroke. Although Flemish and Dutch are very much alike, there are also differences and it is unclear whether the translated questions are also the semantic equivalence in Flemish. This should be investigated further by native-speaking Flemish translators and in a Flemish population in Belgium.

In addition, for adequate international comparison Differential Item Functioning analysis by language is of value to determine whether Dutch- and English-speaking patients answer the ICHOM items differently. We added explanation of some of the medical terms, while this also might be necessary for the comprehensibility of the original questions. Next to the comprehensibility of the questions concerning vascular and systemic medical history, it is unclear how adequate patients answer these questions. Previous research [26] demonstrated that patients do not recall all medical information they have received, suggesting that these questions might be difficult to answer correctly. To overcome this problem, the developers of the original questions have noted that if the patient is unable to answer, this information can be extracted from the medical records. To determine whether and to what extent this is necessary, more experience with the completion of these questions in daily practice is needed.

As for other psychometric properties, test-retest reliability could be assessed. However, the questions are very concrete and therefore no problems regarding reliability are expected. In addition, there might still be bias left through cultural differences, although we have tried to avoid this.

In summary, currently no international standard set for measuring case mix variables and outcomes of stroke patients is used, hampering effective improvement of stroke health care and usability of research. In 2016, the ICHOM has proposed a Standard Set for Stroke for this purpose. The availability of suitable translations of both the single questions and the PROMIS Global Health of this Standard Set for Stroke in different languages will contribute to the international implementation and comparison of stroke outcomes. In this study we have provided a comprehensible translation of the single questions to complete the Dutch PROMs of the ICHOM Standard Set for Stroke.

### Electronic supplementary material

Below is the link to the electronic supplementary material.

**Supplementary Table 1** Translation of the single questions of the ICHOM Standard Set for Stroke.

## Data Availability

All data relevant to the study are included in the article or uploaded as supplementary information.
