# Supplementary material for: Translation and cross-cultural adaptation of the ICHOM standard set for stroke: the Dutch version
Source: J Patient Rep Outcomes. 2023 Sep 11;7:91. doi: 10.1186/s41687-023-00630-7 (PMC10495300; doi:10.1186/s41687-023-00630-7)
Supplement: Supplementary file 1 — Supplementary Table 1 Translation of the single questions of the ICHOM Standard Set for Stroke. [file 41687_2023_630_MOESM1_ESM.docx]

**Supplementary Table 1** Translation of the single questions of the ICHOM Standard Set for Stroke

| Variable  (Variable ID) | Original Question ICHOM | Translation into Dutch by translator 1 | Translation into Dutch by translator 2 | Consensus Dutch Translation | Pre-final Dutch Translation  (after adjustments based on back-translations) |
| --- | --- | --- | --- | --- | --- |
| **Demographic factors** | | | | | |
| Ethnicity  (ETHNIC)^1^ | Varies by country and should be determined by country (not for cross country  comparison) | In welk land ben u geboren?  0 = in Nederland  1 = in een Westers land anders dan Nederland (o.a. de landen van de Europese Unie)  2 = in een niet-Westers land (o.a. Marokko, Turkije, Suriname etc)  3 = anders, namelijk: …  In welk land zijn uw biologische ouders geboren?  0 = beiden zijn in Nederland geboren  1 = een of beiden zijn in een Westers land anders dan Nederland (o.a. de landen van de Europese Unie) geboren  2 = een of beiden zijn in een niet-Westers land (o.a. Marokko, Turkije, Suriname etc) geboren  3 = anders, namelijk: … | In welk land bent u geboren?  In welk land is uw vader geboren?  In welk land is uw moeder geboren? | In welk land bent u geboren?  In welk land is uw biologische vader geboren?  In welk land is uw biologische moeder geboren? | In welk land bent u geboren?  In welk land is uw biologische vader geboren?  In welk land is uw biologische moeder geboren? |
| Living location pre index event (LIVINGLOCPRE)^1^ | Where were you living prior to your stroke or transient ischaemic attack (TIA)?  1 = At home, with no community support  2 = At home with community support  3 = In an assisting living home in the community (senior's home)  4 = In a rehabilitation hospital or skilled care facilities (SNIF, IRF, LTACH)  5 = In long term care (nursing home, chronic care hospital)  888 = Other  999 = Unknown | Waar verbleef u voordat u een beroerte of TIA kreeg?  1 = Thuis zonder thuiszorg  2 = Thuis met hulp van thuiszorg  3 = In een aanleunwoning  4 = In een revalidatiecentrum, in een zorghotel of een revalidatieafdeling in een verpleeghuis  5 = Op een verblijfsafdeling in een verpleeghuis  888 = Anders  999 = Onbekend | Waar woonde u voor uw beroerte of TIA?  1 = Thuis, zonder steun van de gemeenschap  2 = Thuis met steun van de gemeenschap  3 = In een begeleid wonen huis  4 = In een revalidatiecentrum of verpleeghuis  5 = In een instelling voor chronische zorg (verzorgingshuis)  888 = Ander  999 = Onbekend | Waar verbleef u voordat u een beroerte of TIA kreeg?  1 = Thuis zonder hulp van thuiszorg, familie of vrienden  2 = Thuis met hulp van thuiszorg, familie of vrienden  3 = In een aanleunwoning  4 = In een revalidatiecentrum, in een zorghotel of een revalidatieafdeling in een verpleeghuis  5 = Op een verblijfsafdeling in een verpleeghuis  888 = Anders  999 = Onbekend | Waar verbleef u voordat u een beroerte of TIA kreeg?    1 = Thuis zonder hulp van thuiszorg en/of buurtzorg  2 = Thuis met hulp van thuiszorg en/of buurtzorg  3 = In een aanleunwoning, woonzorgcentrum of focuswoning  4 = In een revalidatiecentrum, in een zorghotel of een revalidatieafdeling in een verpleeghuis  5 = Op een verblijfsafdeling in een verpleeghuis  888 = Anders  999 = Onbekend  *LIVINGLOCREHAB*  *Indien u antwoord 4 heeft gegeven: waar woonde u voordat u in een revalidatiecentrum, in een zorghotel of een revalidatieafdeling in een verpleeghuis was opgenomen?*  *1 = Thuis zonder hulp van thuiszorg en/of buurtzorg*  *2 = Thuis met hulp van thuiszorg en/of buurtzorg*  *3 = In een aanleunwoning, woonzorgcentrum of focuswoning*  *5 = Op een verblijfsafdeling in een verpleeghuis*  *888 = Anders*  *999 = Onbekend* |
| Living location post index event (LIVINGLOCPOST)^3^ | Where are you living now?  1 = At home, with no community support  2 = At home with community support  3 = In an assisting living home in the community (senior's home)  4 = In a rehabilitation hospital or skilled care facilities (SNIF, IRF, LTACH)  5 = In long term care (nursing home, chronic care hospital  6 = In an acute care hospital  888 = Other  999 = Unknown | Waar verblijft u momenteel?  1 = Thuis zonder thuiszorg  2 = Thuis met hulp van thuiszorg  3 = In een aanleunwoning  4 = In een revalidatiecentrum, in een zorghotel of een revalidatieafdeling in een verpleeghuis  5 = Op een verblijfsafdeling in een verpleeghuis  6 = in een ziekenhuis  888 = Anders  999 = Onbekend | Waar woont u nu?  1 = Thuis, zonder steun van de gemeenschap  2 = Thuis met steun van de gemeenschap  3 = In een begeleid wonen huis  4 = In een revalidatiecentrum of verpleeghuis  5 = In een instelling voor chronische zorg (verzorgingshuis)  6 = in een ziekenhuis  888 = Ander  999 = Onbekend | Waar verblijft u op dit moment?  1 = Thuis zonder hulp van thuiszorg, familie of vrienden  2 = Thuis met hulp van thuiszorg, familie of vrienden  3 = In een aanleunwoning  4 = In een revalidatiecentrum, in een zorghotel of een revalidatieafdeling in een verpleeghuis  5 = Op een verblijfsafdeling in een verpleeghuis  6 = in een ziekenhuis  888 = Anders  999 = Onbekend | Waar verblijft u op dit moment?  1 = Thuis zonder hulp van thuiszorg en/of buurtzorg  2 = Thuis met hulp van thuiszorg en/of buurtzorg  3 = In een aanleunwoning, woonzorgcentrum of focuswoning  4 = In een revalidatiecentrum, in een zorghotel of een revalidatieafdeling in een verpleeghuis  5 = Op een verblijfsafdeling in een verpleeghuis  6 = in een ziekenhuis  888 = Anders  999 = Onbekend |
| Living alone pre-index event  (LIVEALONEPRE)^1^  If "1 = At home, with no community support" | Did you live alone prior to your stroke or transient ischaemic attack (TIA)?  1 = Yes, I lived alone  2 = No, I shared my household with spouse/partner or other person (e.g. sibling,  children, parents)  999 = Unknown | Woonde u alleen voordat u een beroerte of TIA kreeg?  1 = Ja, ik woonde alleen  2 = Nee, ik woonde samen met mijn partner en/of andere personen (zoals kinderen, broers, zussen, ouders)  999 = Onbekend | Woonde u alleen voor uw beroerte of TIA?  1 = Ja, ik woonde alleen  2 = Nee, ik deelde mijn huishouden met mijn echtgenoot/partner of een ander persoon (bijvoorbeeld broer of zus, kinderen, ouders)  999 = Onbekend | Woonde u alleen voordat u een beroerte of TIA kreeg?  1 = Ja, ik woonde alleen  2 = Nee, ik woonde samen met mijn partner en/of andere personen (zoals kinderen, broers, zussen, ouders)  999 = Onbekend | Woonde u alleen voordat u een beroerte of TIA kreeg?    1 = Ja, ik woonde alleen  2 = Nee, ik woonde samen met mijn partner en/of andere personen (zoals kinderen, broers, zussen, ouders)  999 = Onbekend |
| Living alone post-index event (LIVEALONEPOST)^3^ | Do you live alone now?  1 = Yes, I live alone  2 = No, I share my household with spouse/partner or other person (e.g. sibling, children, parents)  999 = Unknown | Woont u momenteel alleen?  1 = Ja, ik woon alleen  2 = Nee, ik woon samen met mijn partner en/of andere personen (zoals kinderen, broers, zussen, ouders)  999 = Onbekend | Woont u alleen op dit moment?  1 = Ja, ik woon alleen  2 = Nee, ik deel mijn huishouden met mijn echtgenoot/partner of een ander persoon (bijvoorbeeld broer of zus, kinderen, ouders)  999 = Onbekend | Woont u alleen op dit moment?  1 = Ja, ik woon alleen  2 = Nee, ik woon samen met mijn partner en/of andere personen (zoals kinderen, broers, zussen, ouders)  999 = Onbekend | Woont u alleen op dit moment?  1 = Ja, ik woon alleen  2 = Nee, ik woon samen met mijn partner en/of andere personen (zoals kinderen, broers, zussen, ouders)  999 = Onbekend |
| Prestroke functional status – Ambulation  (PRESTROKEAMB)^1^ | Were you able to walk prior to your stroke or transient ischaemic attack (TIA)?  1 = Able to walk without help from another person with or without a device  2 = Able to walk with help from another person  3 = Unable to walk | Kon u lopen voordat u een beroerte of TIA kreeg?  1 = Ik kon lopen zonder hulp van een persoon of hulpmiddel  2 = ik kon lopen met hulp van een persoon  3 = ik kon niet lopen | Was u in staat om te lopen voor uw beroerte of TIA?  1 = in staat om te lopen zonder hulp van iemand anders met of zonder hulpmiddel  2 = in staat om te lopen met hulp van iemand anders  3 = niet in staat om te lopen | Kon u lopen voordat u een beroerte of TIA kreeg?  1 = Ik kon lopen zonder hulp van een persoon of hulpmiddel  2 = Ik kon lopen met hulp van een persoon  3 = Ik kon niet lopen | Kon u lopen voordat u een beroerte of TIA kreeg?  1 = Ik kon lopen zonder hulp van een persoon *met of zonder* hulpmiddel  2 = ik kon lopen met hulp van een persoon *met of zonder* hulpmiddel  3 = ik kon niet lopen |
| Prestroke functional status – Toileting  (PRESTROKETOILET)^1^ | Did you need help from anybody to go to the toilet prior to your stroke or transient  ischaemic attack (TIA)?  1 = I could manage going to the toilet without assistance  2 = I needed help to go to the toilet | Had u hulp nodig om naar toilet te gaan voordat u een beroerte of TIA kreeg?  1 = Ik kon zelfstandig naar toilet  2 = Ik had hulp nodig om naar toilet te gaan | Had u hulp nodig van iemand anders om naar het toilet te gaan voor uw beroerte of TIA?  1 = Ik kon naar het toilet gaan zonder hulp  2 = Ik had hulp nodig om naar het toilet te gaan | Had u van iemand hulp nodig om naar het toilet te gaan voordat u een beroerte of TIA kreeg?  1 = Ik kon zelfstandig naar het toilet  2 = Ik had hulp nodig om naar het toilet te gaan | Had u van iemand  hulp nodig om naar het toilet te gaan voordat u een beroerte of TIA kreeg?  1 = Ik kon zelfstandig naar het toilet  2 = Ik had hulp nodig om naar het toilet te gaan |
| Prestroke functional status – Dressing  (PRESTROKEDRESS)^1^ | Did you need help with dressing/undressing prior to your stroke or transient ischaemic attack (TIA)?  1 = I could manage dressing/undressing without help  2 = I needed help dressing/undressing | Had u hulp nodig bij aan- en uitkleden voordat u een beroerte of TIA kreeg?  1 = Ik kon mijzelf aan- en uitkleden zonder hulp  2 = Ik had hulp nodig bij aan- en uitkleden | Had u hulp nodig met aan- en uitkleden voor uw beroerte of TIA?  1 = ik kon aan- en uitkleden zonder hulp  2 = ik had hulp nodig met aan- en uitkleden | Had u hulp nodig bij aan- en uitkleden voordat u een beroerte of TIA kreeg?  1 = Ik kon mijzelf aan- en uitkleden zonder hulp  2 = Ik had hulp nodig bij aan- en uitkleden | Had u hulp nodig bij aan- en uitkleden voordat u een beroerte of TIA kreeg?    1 = Ik kon mijzelf aan- en uitkleden zonder hulp  2 = Ik had hulp nodig bij aan- en uitkleden |
| **Vascular and Systemic**  All these items are phrased as a patient reported measure. However, if the patient is unable to answer, this information can be abstracted from the medical records. | | | | | |
| Prior Stroke (PRIORSTROKE)^1^ | Prior to this hospitalization, have you ever been told by a doctor that you have had a stroke?  0 = No  1 = Yes  999 = Unknown | Heeft u voor de opname voor deze beroerte ooit eerder van uw dokter gehoord dat u een beroerte heeft gehad?  0 = Nee  1 = Ja  999 = Ik weet het niet | Heeft een arts u voor deze ziekenhuisopname ooit verteld dat u een beroerte heeft gehad?  0 = Nee  1 = Ja  999 = Onbekend | Heeft een dokter u voor deze ziekenhuisopname voor de beroerte ooit eerder verteld dat u een beroerte heeft gehad?  0 = Nee  1 = Ja  999 = Ik weet het niet | Heeft een dokter u voor deze ziekenhuisopname voor de beroerte ooit eerder verteld dat u een beroerte heeft gehad?  0 = Nee  1 = Ja  999 = Ik weet het niet |
| Prior TIA  (PRIORTIA)^1^ | Have you ever been told by a doctor that you have had a transient ischemic attack (this is sometimes called a TIA or mini-stroke)?  0 = No  1 = Yes  999 = Unknown | Heeft u ooit van uw dokter gehoord dat u een TIA heeft gehad?  0 = Nee  1 = Ja  999 = Ik weet het niet | Heeft een arts u ooit verteld dat u een TIA (dit wordt ook wel een kleine beroerte genoemd) heeft gehad?  0 = Nee  1 = Ja  999 = Onbekend | Heeft een dokter u ooit verteld dat u een TIA heeft gehad?    0 = Nee  1 = Ja  999 = Ik weet het niet | Heeft een dokter u ooit verteld dat u een TIA heeft gehad?    0 = Nee  1 = Ja  999 = Ik weet het niet |
| Prior MI  (PRIORMI)^1^ | Have you ever been told by your doctor that you've had a heart attack (this is sometimes called a myocardial infarction, or MI)?  0 = No  1 = Yes  999 = Unknown | Heeft u ooit van uw dokter gehoord dat u een hartaanval heeft gehad (ook wel myocardinfarct genoemd)?  0 = Nee  1 = Ja  999 = Ik weet het niet | Heeft een arts u ooit verteld dat u een hartaanval (dit wordt ook wel een myocardinfarct genoemd) heeft gehad?  0 = Nee  1 = Ja  999 = Onbekend | Heeft een dokter u ooit verteld dat u een hartaanval heeft gehad (ook wel myocardinfarct genoemd)?  0 = Nee  1 = Ja  999 = Ik weet het niet | Heeft een dokter u ooit verteld dat u een hartaanval heeft gehad?  0 = Nee  1 = Ja  999 = Ik weet het niet |
| Coronary artery disease  (CAD)^1^ | Have you ever been told by your doctor that you have coronary artery disease?  0 = No  1 = Yes  999 = Unknown | Heeft u ooit van uw dokter gehoord dat u coronaire hartziekten heeft (ook wel angina pectoris genoemd)?  0 = Nee  1 = Ja  999 = Ik weet het niet | Heeft een arts u ooit verteld dat u een coronaire hartziekte heeft?  0 = Nee  1 = Ja  999 = Onbekend | Heeft een dokter u ooit verteld dat u coronaire hartziekten heeft (ook wel angina pectoris genoemd)?  0 = Nee  1 = Ja  999 = Ik weet het niet | Heeft een dokter u ooit verteld dat u coronaire hartziekten heeft (ook wel angina pectoris genoemd)?  0 = Nee  1 = Ja  999 = Ik weet het niet |
| Atrial fibrillation (AFIB)^1^ | Have you ever been told by your doctor that you have atrial fibrillation?  0 = No  1 = Yes  999 = Unknown | Heeft u ooit van uw dokter gehoord dat u boezemfibrilleren heeft (ook wel atriumfibrilleren genoemd)?  0 = Nee  1 = Ja  999 = Ik weet het niet | Heeft een arts u ooit verteld dat u atriumfibrilleren (dit wordt ook wel boezemfibrilleren genoemd) heeft?  0 = Nee  1 = Ja  999 = Onbekend | Heeft een dokter u ooit verteld dat u boezemfibrilleren heeft (ook wel atriumfibrilleren genoemd)?  0 = Nee  1 = Ja  999 = Ik weet het niet | Heeft een dokter u ooit verteld dat u boezemfibrilleren heeft (ook wel atriumfibrilleren genoemd)?  0 = Nee  1 = Ja  999 = Ik weet het niet |
| Diabetes mellitus (DIAB)^1^ | Have you ever been told by your doctor that you have diabetes?  0 = No  1 = Yes  999 = Unknown | Heeft u ooit van uw dokter gehoord dat u suikerziekte heeft (ook wel diabetes genoemd)?  0 = Nee  1 = Ja  999 = Ik weet het niet | Heeft een arts u ooit verteld dat u diabetes heeft?  0 = Nee  1 = Ja  999 = Onbekend | Heeft een dokter u ooit verteld dat u suikerziekte heeft (ook wel diabetes genoemd)?  0 = Nee  1 = Ja  999 = Ik weet het niet | Heeft een dokter u ooit verteld dat u suikerziekte heeft (ook wel diabetes genoemd)?  0 = Nee  1 = Ja  999 = Ik weet het niet |
| Hypertension (HYPERTENS)^1^ | Have you ever been told by a doctor that you have high blood pressure (this is  sometimes called hypertension)?  0 = No  1 = Yes  999 = Unknown | Heeft u ooit van uw dokter gehoord dat u een verhoogde bloeddruk heeft (ook wel hypertensie genoemd)?  0 = Nee  1 = Ja  999 = Ik weet het niet | Heeft een arts u ooit verteld dat u hoge bloeddruk (dit wordt ook wel hypertensie genoemd) heeft?  0 = Nee  1 = Ja  999 = Onbekend | Heeft een dokter u ooit verteld dat u een hoge bloeddruk heeft (ook wel hypertensie genoemd)?  0 = Nee  1 = Ja  999 = Ik weet het niet | Heeft een dokter u ooit verteld dat u een hoge bloeddruk heeft (ook wel hypertensie genoemd)?  0 = Nee  1 = Ja  999 = Ik weet het niet |
| Hyperlipidemia (HYPERLIP)^1^ | Have you ever been told by your doctor that you have high cholesterol (this is sometimes called hyperlipidemia or dyslipidemia)?  0 = No  1 = Yes  999 = Unknown | Heeft u ooit van uw dokter gehoord dat u een verhoogd cholesterol heeft (ook wel hypercholesterolemie genoemd)?  0 = Nee  1 = Ja  999 = Ik weet het niet | Heeft een arts u ooit verteld dat u hoog cholesterol (dit wordt ook wel hyperlipidemie of dyslipidemie genoemd) heeft?  0 = Nee  1 = Ja  999 = Onbekend | Heeft een dokter u ooit verteld dat u een hoog cholesterol heeft?  0 = Nee  1 = Ja  999 = Ik weet het niet | Heeft een dokter u ooit verteld dat u een hoog cholesterol heeft?  0 = Nee  1 = Ja  999 = Ik weet het niet |
| Smoking status (SMOKE)^1^ | Do you currently smoke, or have you smoked cigarettes or tobacco over the past year?  0 = No  1 = Yes  999 = Unknown | Rookt u of heeft u in het afgelopen jaar gerookt?  0 = Nee  1 = Ja  999 = Onbekend | Rookt u op dit moment of heeft u sigaretten of tabak gerookt in het afgelopen jaar?  0 = Nee  1 = Ja  999 = Onbekend | Rookt u op dit moment of heeft u het afgelopen jaar gerookt?  0 = Nee  1 = Ja  999 = Onbekend | Rookt u op dit moment of heeft u  het afgelopen jaar gerookt?  0 = Nee  1 = Ja  999 = Onbekend |
| Alcohol use (ALCOHOL)^1^ | Do you drink more than one alcoholic drink a day?  0 = No  1 = Yes  999 = Unknown | Drinkt u meer dan 1 glas alcohol per dag?  0 = Nee  1 = Ja  999 = Onbekend | Drinkt u meer dan 1 alcoholisch drankje per dag?  0 = Nee  1 = Ja  999 = Onbekend | Drinkt u meer dan 1 glas alcohol per dag?  0 = Nee  1 = Ja  999 = Onbekend | Drinkt u meer dan 1 glas alcohol per dag?  0 = Nee  1 = Ja  999 = Onbekend |
| **Survival and Disease controle** | | | | | |
| Report of new stroke within 90 days after admission for stroke (STROKERECUR)^3^ | After your hospitalization for stroke, have you been told by a doctor that you have had a new stroke?  0 = No  1 = Yes  999 = Unknown | Heeft u van een dokter gehoord dat u sinds uw beroerte opnieuw een beroerte heeft gehad?  0 = Nee  1 = Ja  999 = Ik weet het niet | Na uw ziekenhuisopname voor de beroerte, heeft een arts u verteld dat u opnieuw een beroerte heeft gehad?  0 = Nee  1 = Ja  999 = Onbekend | Na de ziekenhuisopname voor uw beroerte, heeft een dokter u verteld dat u opnieuw een beroerte heeft gehad?  0 = Nee  1 = Ja  999 = Ik weet het niet | Na de ziekenhuisopname voor uw beroerte, heeft een dokter u verteld dat u opnieuw een beroerte heeft gehad?  0 = Nee  1 = Ja  999 = Ik weet het niet |
| Smoking cessation (SMOKECESS)^3^  If "1 = Yes" to SMOKE | Since your hospitalization for stroke, have you smoked tobacco or cigarettes?  0 = No  1 = Yes  999 = Unknown | Bent u sinds uw beroerte gestopt met roken?  0 = Nee  1 = Ja  999 = Onbekend | Heeft u sigaretten of tabak gerookt na uw ziekenhuisopname voor uw beroerte?  0 = Nee  1 = Ja  999 = Onbekend | Heeft u gerookt na de ziekenhuisopname voor uw beroerte?  0 = Nee  1 = Ja  999 = Onbekend | Heeft u gerookt na uw beroerte?  0 = Nee  1 = Ja  999 = Onbekend |
| **Patient-Reported Health Status** | | | | | |
| Poststroke functional status – Ambulation (POSTSTROKEAMB)^2,3^ | Are you able to walk?  1 = Able to walk without help from another person with or without a device  2 = Able to walk with help from another person  3 = Unable to walk | Kan u lopen?  1 = Ik kan lopen zonder hulp van een persoon of hulpmiddel  2 = Ik kan lopen met hulp van een persoon  3 = Ik kan niet lopen | Bent u in staat om te lopen?  1 = In staat om te lopen zonder hulp van iemand anders met of zonder hulpmiddel  2 = In staat om te lopen met hulp van iemand anders  3 = Niet in staat om te lopen | Kunt u lopen?  1 = Ik kan lopen zonder hulp van een persoon of hulpmiddel  2 = Ik kan lopen met hulp van een persoon  3 = Ik kan niet lopen | Kunt u lopen?  1 = Ik kan lopen zonder hulp van een persoon *met of zonder* hulpmiddel  2 = Ik kan lopen met hulp van een persoon *met of zonder* hulpmiddel  3 = Ik kan niet lopen |
| Poststroke functional status – Toileting (POSTSTROKETOILET)^2,3^ | Do you need help from anybody to go to the toilet?  1 = I can manage going to the toilet without assistance  2 = I need help to go to the toilet | Heeft u hulp nodig om naar toilet te gaan?  1 = Ik kan zelfstandig naar toilet  2 = Ik heb hulp nodig om naar toilet te gaan | Heeft u hulp nodig van iemand anders om naar het toilet te gaan?  1 = Ik kan naar het toilet gaan zonder hulp  2 = Ik heb hulp nodig om naar het toilet te gaan | Heeft u van iemand hulp nodig om naar het toilet te gaan?  1 = Ik kan zelfstandig naar het toilet  2 = Ik heb hulp nodig om naar het toilet te gaan | Heeft u van iemand hulp nodig om naar het toilet te gaan?  1 = Ik kan zelfstandig naar het toilet  2 = Ik heb hulp nodig om naar het toilet te gaan |
| Poststroke functional status – Dressing (POSTSTROKEDRESS)^2,3^ | Do you need help with dressing/undressing?  1 = I can manage dressing/undressing without help  2 = I need help dressing/undressing | Heeft u hulp nodig bij aan- en uitkleden?  1 = Ik kan mijzelf aan- en uitkleden zonder hulp  2 = Ik heb hulp nodig bij aan- en uitkleden | Heeft u hulp nodig met aan- en uitkleden?  1 = Ik kan aan- en uitkleden zonder hulp  2 = Ik heb hulp nodig met aan- en uitkleden | Heeft u hulp nodig bij aan- en uitkleden?  1 = Ik kan mijzelf aan- en uitkleden zonder hulp  2 = Ik heb hulp nodig bij aan- en uitkleden | Heeft u hulp nodig bij aan- en uitkleden?  1 = Ik kan mijzelf aan- en uitkleden zonder hulp  2 = Ik heb hulp nodig bij aan- en uitkleden |
| Feeding (FEEDING)^2,3^ | Do you need a tube for feeding?  0 = No  1 = Yes | Heeft u een sonde nodig om te eten, b.v. een neussonde of een maagsonde?  0 = Nee  1 = Ja | Heeft u een sonde nodig om te eten?  0 = Nee  1 = Ja | Heeft u een sonde nodig om te eten, b.v. een neussonde of een maagsonde?  0 = Nee  1 = Ja | Heeft u een sonde nodig om te eten, b.v. een neussonde of een maagsonde?  0 = Nee  1 = Ja |
| Ability to communicate (COMMUNIC)^2,3^ | Do you have problems with communication or understanding?  0 = No  1 = Yes | Heeft u problemen met praten of begrijpen van taal?  0 = Nee  1 = Ja | Heeft u problemen met communiceren of het begrijpen van communicatie?  0 = Nee  1 = Ja | Heeft u problemen met praten of begrijpen van taal?  0 = Nee  1 = Ja | Heeft u problemen met praten of begrijpen van taal?  0 = Nee  1 = Ja |

^1^Timing question: Admission for index event

^2^Timing question: Discharge + 7 days

^3^Timing question: 90 days post admission for index event
